# Mechanistic screening of reproductive toxicity in a novel 3D testicular co-culture model shows significant impairments following exposure to low-dibutyl phthalate concentrations

**DOI:** 10.1007/s00204-024-03767-6

**Published:** 2024-05-20

**Authors:** Radwa Almamoun, Paula Pierozan, Oskar Karlsson

**Affiliations:** Science for Life Laboratory, Department of Environmental Science, Stockholm University, 11418 Stockholm, Sweden

**Keywords:** Antiandrogen, Reproductive toxicity, Mechanistic toxicology, Minipuberty, Spermatogenesis, Testosterone

## Abstract

To improve the mechanistic screening of reproductive toxicants in  chemical-risk assessment and drug development, we have developed a three-dimensional (3D) heterogenous testicular co-culture model from neonatal mice. Di-n-butyl phthalate (DBP), an environmental contaminant that can affect reproductive health negatively, was used as a model compound to illustrate the utility of the in vitro model. The cells were treated with DBP (1 nM to 100 µM) for 7 days. Automated high-content imaging confirmed the presence of cell-specific markers of Leydig cells (CYP11A1 +), Sertoli cells (SOX9 +), and germ cells (DAZL +). Steroidogenic activity of Leydig cells was demonstrated by analyzing testosterone levels in the culture medium. DBP induced a concentration-dependent reduction in testosterone levels and decreased the number of Leydig cells compared to vehicle control. The levels of steroidogenic regulator StAR and the steroidogenic enzyme CYP11A1 were decreased already at the lowest DBP concentration (1 nM), demonstrating upstream effects in the testosterone biosynthesis pathway. Furthermore, exposure to 10 nM DBP decreased the levels of the germ cell-specific RNA binding protein DAZL, central for the spermatogenesis. The 3D model also captured the development of the Sertoli cell junction proteins, N-cadherin and Zonula occludens protein 1 (ZO-1), critical for the blood–testis barrier. However, DBP exposure did not significantly alter the cadherin and ZO-1 levels. Altogether, this 3D in vitro system models testicular cellular signaling and function, making it a powerful tool for mechanistic screening of developmental testicular toxicity. This can open a new avenue for high throughput screening of chemically-induced reproductive toxicity during sensitive developmental phases.

## Introduction

The male reproductive system is susceptible to endocrine disruption during key stages of its development (Wohlfahrt-Veje et al. 2009). Over the past two decades, various trends of adverse effects in male reproductive health have been highlighted, which include rising incidence rate of testicular cancer, declining semen quality and genital malformations (Björndahl et al. 2022; Skakkebæk et al. 2022). Exposure to environmental toxicants has been suggested as a contributing factor for this increase (Mann et al. 2020; Wohlfahrt-Veje et al. 2009). In particular, there is a high interest in the potential adverse effects of endocrine disrupting chemicals (EDCs), such as phthalates, on the reproductive system (Sharpe and Skakkebaek 1993). To better assess and understand the potential adverse effects of EDCs on the male reproductive system, more mechanistic in vitro studies are warranted. Several in vitro testicular models have been developed for investigating various aspects of testicular development, function, disease, and toxicology. These include two-dimensional models (2D) such as mono-culture of testicular cells (e.g., Leydig cell, germ cell or Sertoli cells) or co-culture of several populations of isolated cells. Three-dimensional (3D) models incorporate culture of dissociated testicular cells or small tissue fragments (known as testicular organ-culture) in supportive 3D matrix systems, providing a beneficial effect on cell–to–cell interaction. The use of primary testicular in vitro models, like the current 3D co-culture, can advance our understanding of Leydig cell testosterone production and regulation. This model can simulate testicular physiology, which is not possible with the widely used mouse tumor Leydig cell line MA-10, as it does not express CYP17 and mostly produces progesterone instead of testosterone (Engeli et al. 2018; Zirkin and Papadopoulos 2018).

Given the endocrine disrupting properties of phthalates, a large group of widely used manmade chemicals, there is public concern over the potential adverse health effects in early life, particularly during genital development and neurodevelopment (Jurewicz and Hanke 2011). Experimentally, developmental exposure to phthalates, including di-*n*-butyl phthalate (DBP), has been shown to induce a “phthalate syndrome”, which mimics the testicular dysgenesis syndrome (TDS) in humans (Fisher et al. 2003; Skakkebaek et al. 2001; Wohlfahrt-Veje et al. 2009). TDS is hypothesized to be a multifactorial disorder and is characterized by birth genital malformations (e.g., cryptorchidism and hypospadias), impaired spermatogenesis and other genital malformations. For instance, developmental reproductive malformations were found when fetal testosterone production was reduced by 25–70% following in utero exposure to a mixture of phthalates in rats (Howdeshell et al. 2015). One epidemiologic study demonstrated inverse relationships between anogenital distance index (AGI) and incomplete testicular descent with the corresponding maternal levels of four urinary phthalate metabolites: monobutyl phthalate (MBP), monoethyl phthalate (MEP), monobenzyl phthalate (MBzP), and monoisobutyl phthalate (MiBP) (Swan et al. 2005).

Phthalates are widely used in industrial applications as plasticizers, primarily in the manufacturing of flexible polyvinyl chloride (PVC) plastic. DBP is one of the most common phthalic acid ester used in a wide array of products, including adhesives, printing inks, PVC piping, paper coatings, plastic packaging, pharmaceuticals coating, and as solvents in various personal-care products (Hauser and Calafat 2005; Horn et al. 2004). As DBP is not covalently bound to the polymer matrix, it can leach out and contaminate the environment (Gao and Wen 2016). Humans are therefore continuously exposed to phthalates via ingestion, dermal contact and inhalation. DBP and its active metabolite MBP have been extensively detected in different matrices such as blood, urine, amniotic fluid, breast milk, and cord blood (Lien et al. 2015; Watkins et al. 2016). Neonates and infants may be particularly vulnerable due to ongoing critical developmental processes, as well as high exposure levels due to hand-to-mouth activity (Braun et al. 2013; Sathyanarayana 2008). Breastfeeding is considered as an additional exposure route for newborns and infants, as significant levels of phthalates, including DBP, can be excreted in breast milk (Calafat et al. 2004; Latini et al. 2009; Mortensen et al. 2005).

In this study, we have developed a mouse 3D scaffolds testicular co-culture model of germ, Leydig and Sertoli cells that allows assessment of various aspects of male developmental reproductive toxicity. DBP (1 nM – 100 µM) was used as a model compound to illustrate the utility of the in vitro model by characterizing specific testicular cell signaling pathways involved in its reproductive toxicity, focusing on testosterone production and formation of inter-Sertoli tight and adherens junctions.

## Materials and methods

### Chemicals and reagents

DBP (CAS No 84-74-2, purity > 99%), dimethyl sulfoxide (DMSO), paraformaldehyde, 4´,6-diamidino-2-phenylindole dihydrochloride (DAPI), collagenase, hyaluronidase, DNase, Triton X-100, 3-(4,5-dimethyl2-yl)-2,5-diphenyltetrazolium bromide (MTT), nonessential amino acids, epidermal growth factor (EGF), sodium pyruvate, sodium lactate and bovine serum albumin (BSA) were purchased from Sigma Aldrich (St. Louis, MO, USA). Phosphate-Buffered Saline (PBS) and Minimum Essential Medium (MEM no glutamine, no phenol red) were purchased from Gibco (Thermo Fischer Scientific, NY, USA). Tween 20 and NaCl were purchased from Fisher (Loughborough, UK). Penicillin–streptomycin (P/S), trypsin solution (0.05%) and trypsinedta were obtained from Gibco (Life Technologies, Paisley, UK). Insulin Transferrin-Selenium (ITS) premix was purchased from Corning Discovery Labware, Inc. (Bedford, MA, USA). Matrigel Basement Membrane Matrix was obtained from Corning (MA, USA). Anti-SOX9 monoclonal (ab207677), anti-ZO-1 monoclonal (ab221547), anti-N-cadherin monoclonal (ab98952), anti-DAZL monoclonal (ab215718) and normal goat serum (blocking agent, ab7481) were obtained from Abcam (Cambridge, UK). Anti-CYP11A1 (PA5-109,610), Anti-StAR (PA5-21,687), Alexa Fluor 568 Phalloidin (A12380), Goat anti-rabbit IgG Alexa Fluor 488 (A-11008), Goat anti-mouse IgG Alexa Fluor 647 (A-21235), HSD17B3 polyclonal antibody (PA5-30,063), CYP17A1 monoclonal antibody (MA5-35,632), and LHR polyclonal antibody (PA5-97,923) were purchased from Thermo Fisher Scientific (Waltham, MA, USA).

### 3D testicular co-culture and DBP exposure

Pregnant C57Bl/6NCrl mice at 8 weeks of age were purchased from (Charles River, Sulzfeld, Germany) and housed in Makrolon cages in a room controlled for temperature and humidity at the Experimental Core Facility (ECF) at Stockholm University. Tap water and food pellets were provided ad libitum. After birth, male pups at the age of 7-day postnatal (PND 7) were euthanized by decapitation, and the testes were collected. The seminiferous tubules were enzymatically dissociated and dispersed into a single cell suspension using a two-step digestion protocol described by Wegner and colleagues with modifications (Wegner et al. 2013). In brief, to harvest the undescended testes from pups, pressure was applied on each side of the lower abdomen to force testicular descent. A small lateral abdominal incision was made using sterile scissors to remove the testes. The testes were transferred to a cell culture dish containing enough fresh MEM to submerge testes. Next, the testes were decapsulated with sterile fine forceps under a dissecting microscope to tear the tunica albuginea and squeeze the seminiferous tubules out. Once isolated, the aggregated seminiferous tubules were transferred into a new dish of MEM and mechanically desegregated and cut into four to six pieces to give enzymes greater access to tissue. The tissues were washed with MEM and allowed to settle at the bottom of a 15 ml Falcon tube to remove excess blood, which may interfere with the digestive enzymes.

In the first digestion step, the testicular tissues were digested using a mixture of collagenase (1 mg/ml), DNase I (0.001 mg/ml), and hyaluronidase (1 mg/ml) in MEM (enzyme cocktail A) for 20 min at 37 °C. The tube was gently agitated at the midpoint of incubation. The supernatant was removed and the tissues were washed with 10 mL of ice-cold MEM for 10 min. In the second-digestion step, MEM was gently removed and the remaining small tissues were processed in another culture medium supplemented with a mixture of collagenase (1 mg/ml) and DNase I (0.001 mg/ml) (enzyme cocktail B) for 20 min at 37 °C. Subsequently, the enzyme cocktail B was removed and the tissues were incubated in ice-cold MEM for 10 min before reapplying cocktail B for additional 20 min at 37 °C. The supernatant was then removed and the tissues were washed with MEM on ice for 10 min, followed by incubation with 0.05% trypsin EDTA (a volume of 5 × the amount of testicular tissue) for 3 min at 37 °C. Next, the trypsin inhibitor solution, consisting of 0.4% DNase I in soybean trypsin inhibitor 1X, was added to the pellets and centrifuged at 150 g for 5 min. The supernatant was removed and the pellets were resuspended in fresh trypsin inhibitor solution followed by a second round of centrifugation at 150 g for 5 min. Finally, the cell pellet was resuspended in trypsin inhibitor solution before being dispersed with a sterile Pasteur pipette and filtered through a nylon mesh cell strainer.

**Fig. 1:**
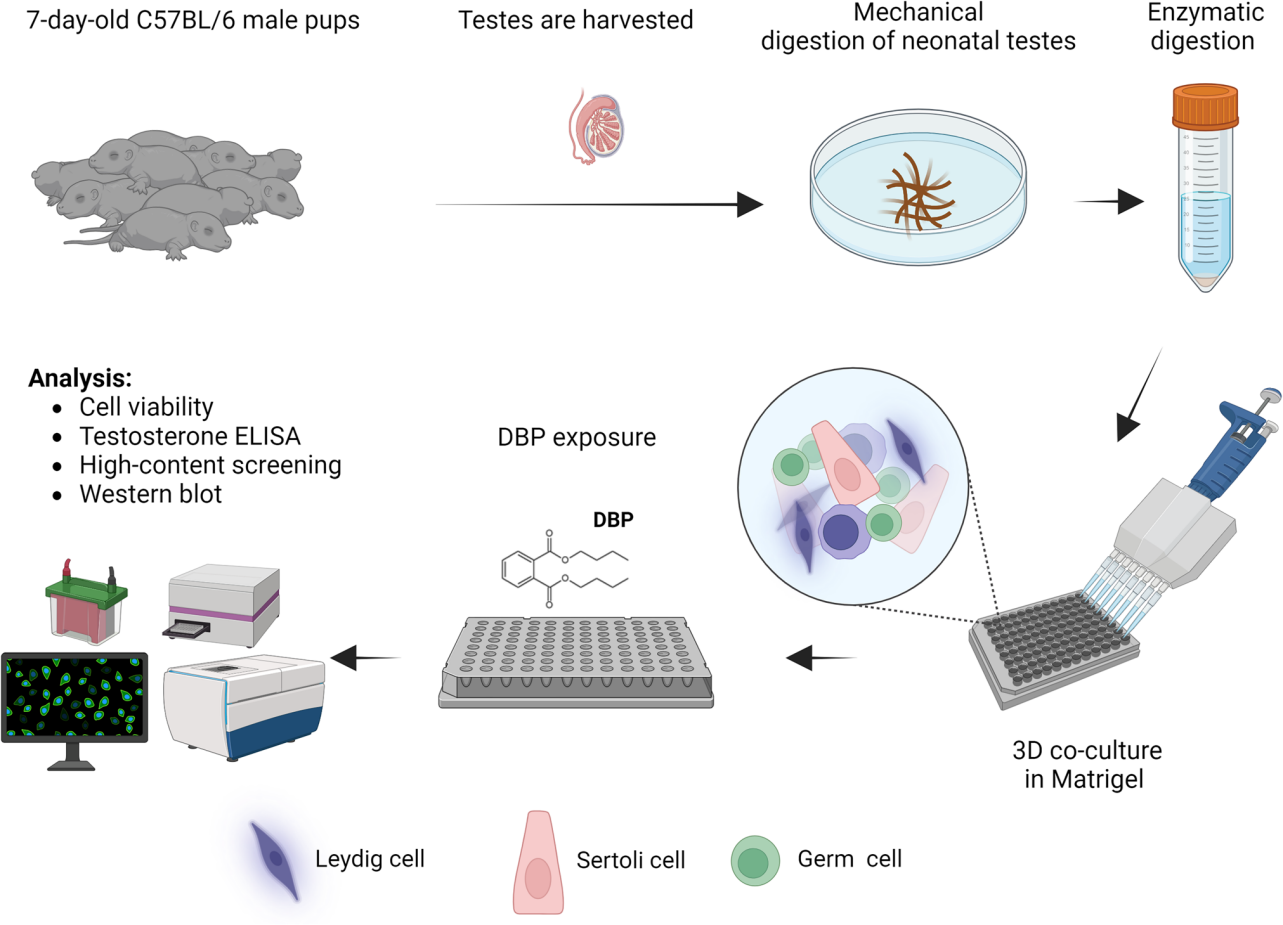
Schematic illustration of the 3D testicular co-culture workflow for mechanistic screening of reproductive toxicants. Di-n-butyl phthalate (DBP) was used as a model compound to illustrate the utility of this in vitro model. Figure created with BioRender.com

The dissociated testicular cells were resuspended in serum-free MEM containing nonessential amino acids (at a final concentration of 0.001 mM), sodium pyruvate (0.01 mM), sodium lactate (0.8 µM), EGF (0.003 µg/ml), penicillin–streptomycin (1% vol/vol) and 1% ITS culture supplement. The viability and the count of the isolated cells were assessed by hemocytomer. After that, cells were seeded in Primaria fibronectin-coated plates (96-well plate or 6-well plate). To prepare fibronectin-coated plates, stock fibronectin solution was diluted in Milli-Q water (1:100) and incubated for 30 min at 4 °C. The fibronectin solution was added to the plates and incubated for at least 2 h at 37 °C. The plates were washed and stored in PBS until ready for cell seeding. For the 96-well plates, the cells were seeded at a density of 4 × 10^4^ cells/well in 100 µl culture media followed by the addition of 15 μl ice-cold Matrigel extracellular matrix overlay (at a final concentration of 150 µg/ml) to the center of each well and gently swirled with cell suspension to facilitate in vivo-like three-dimensional scaffold. For the 6-well plates, the concentration of the cells was 2 × 10^6^ cells/well in 1 ml media. After a 48-h acclimation period, the media in the 3D testicular co-cultures were replaced with new media containing DBP at a range of concentrations, 1 nM, 10 nM, 100 nM, 500 nM, 1 µM, 5 µM, 10 µM, 50 µM and 100 µM for 7 days. Controls were exposed to 0.1% DMSO only. The exposure media were replaced every other day. The culture media were collected at the end of the experiment and stored at  – 80 °C for subsequent ELISA testosterone measurement. A schematic representation of the testicular co-culture workflow is illustrated in Fig. 1.

The animal procedures were approved by the Regional Animal Ethical Committee in accordance with the Swedish legislation on Animal Experimentation (Animal welfare act SFS1998:56) and the European Union Directive on the Protection of Animals Used for Scientific Purposes (2010/63/EU).

### Cell viability analysis

After the DBP treatment, the cell viability was measured by the MTT assay. The culture media was replaced with 100 µl/well of 0.5 mg/ml MTT reagent dissolved in media. After 3 h incubation at 37 °C, the MTT solution was removed, and the generated formazan crystals were solubilized by adding 50 µL DMSO to each well. The absorbance of formazan was measured at 590 nm using a SpectraMax i3 microplate reader (Molecular Devices, San Jose, CA, USA).

### Testosterone analysis

Testosterone levels were measured in the collected cell culture media using a colorimetric competitive enzyme-linked immunosorbent ELISA kit (Enzo Life Sciences, Farmingdale, NY, USA) in accordance with the manufacturer’s instructions. The absorbance was measured at 405 nm using a SpectraMax i3 microplate reader (Molecular Devices, San Jose, CA, USA).

### Immunofluorescence staining of cell-specific markers and key testicular proteins

To analyze protein levels by immunofluorescence and high-content imaging, the co-cultures were washed with PBS and fixed with 4% paraformaldehyde for 20 min. For permeabilization, the cells were incubated with 0.25% Triton X-100 for 10 min followed by three times washing with PBS for 5 min. The cells were then blocked for 3 h in blocking buffer containing 10% normal goat serum, 1% BSA and 0.05% Tween 20 diluted in PBS. Primary antibodies against key testicular proteins were incubated in 1% BSA diluted in PBS overnight at 4 °C. Cells were washed three times in PBS and then incubated with secondary antibodies conjugated with 488, 555 or 647 fluorophores diluted in PBS for 1 h at room temperature. DAPI (0.5 µg/ml) was used for nuclear counterstaining. The total cell count was determined by DAPI staining of all nuclei. The analysis images were acquired using a 10 × air objective on ImageXpress micro XLS confocal High-content analysis system (Molecular devices, Sunnyvale, CA, USA). Nine sites per well were analyzed with MetaXpress Software (Molecular devices, Sunnyvale, CA, USA) after digital acquisition using the cell scoring module. Representative images for the figures were taken using 20 × water immersion objective.

To evaluate the impact of DBP on the main testicular cell populations, the cells were stained with cell-specific antibodies, including RNA splicing and binding protein (anti-DAZL; germ-cell marker), cholesterol side-chain cleavage enzyme (anti-CYP11A1; Leydig cell marker) and transcription factor SOX9 (anti-SOX9; Sertoli cells). The cells were co-stained for SOX9 and CYP11A1. The cells were also stained for the steroidogenic acute regulatory protein (StAR). To investigate the effects of DBP on the expression pattern of junctional proteins in Sertoli cells, the 3D testicular co-culture was triple-immunostained for Cadherin-2 (N-Cadherin), Zonula occludens protein 1 (ZO-1) and F-Actin. The fluorescence intensity was quantified using the cell scoring module, integrated fluorescence application mode via MetaXpress high-content image acquisition and analysis software. The number of cell-marker positive cells divided by DAPI total cells was also analyzed to determine the DBP effect on the relative number of each cell type.

### Western blot analysis of steroidogenic proteins

To analyze protein levels by western blot cells were washed with PBS and trypsinized, and the detached cells were collected and lysed with RIPA buffer (150 mM NaCl, 0.1% Triton X-100, 0.5% Sodium Deoxycholate, 0.1% SDS, 50 mM Tris–HCl, 1% protease inhibitors, pH 8). The protein concentration was determined by Lowry assay. Western blot was performed as previously described (Källsten et al. 2022b). Primary antibodies were diluted at 1:1000 and incubated overnight. The blots were subsequently incubated for 1 h with peroxidase conjugated anti-mouse or anti-rabbit diluted at 1:5000. The blot was developed with a chemiluminescence ECL kit with a charge-coupled device (CCD) imager (iBright CL750 Imaging System, ThermoFisher, Rockford, IL, USA), and the optical density was measured with ImageJ software (NIH, USA). The loading control β-actin was used for normalization and the protein levels are shown as percentage of control.

### Statistical analysis

All the experimental data are expressed as means ± standard error of the mean (SEM). One-way analysis of variance (ANOVA) followed by Dunnett’s multiple comparison test was applied to analyze differences between the DBP-treated cells and controls. The statistics tests were conducted using GraphPad Prism 8 software (San Diego, CA, USA). The difference is considered to be statistically significant when *p*-value < 0.05.

## Results

### Effects of DBP on testicular cells viability and cell number

**Fig. 2:**
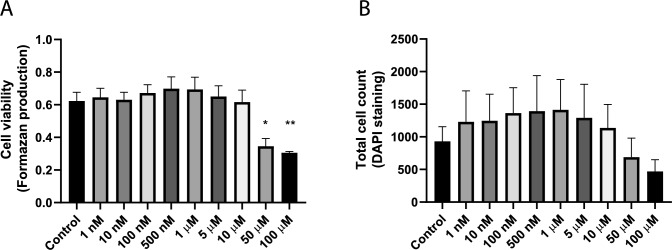
DBP exposure decreased the cell viability of the 3D testicular co-culture at higher concentrations. The cell viability and total cell count were measured following 7-day exposure to either solvent control (0.1% DMSO) or various concentrations of DBP. **A** Cell viability assessed by MTT assay. **B** Total cell count quantified by DAPI-nuclei staining. Values represent mean ± SEM from three experiments. Each treatment condition was performed in three wells per experiment. Statistically significant differences from control are indicated as follows: *p < 0.05 and **p < 0.01 (one-way ANOVA followed by Dunnett’s multiple comparison test)

To assess DBP cytotoxicity and the total cell number, MTT formazan production and DAPI nuclei count were conducted after 7 days of exposure to various DBP concentrations or solvent control (0.1% DMSO). The MTT results demonstrated a 45% and 51% decrease in formazan production after 50 µM (*p* = 0.014) and 100 µM (*p* = 0.004) DBP exposure compared to control, respectively, indicating cytotoxicity at the two highest concentrations (Fig. 2A). The cell count revealed a trend of decreased cell number at the two highest concentrations, 50 µM and 100 µM DBP, but the decrease did not reach statistical significance (Fig. 2B).

### DBP exposure decreased testosterone production in vitro

**Fig. 3:**
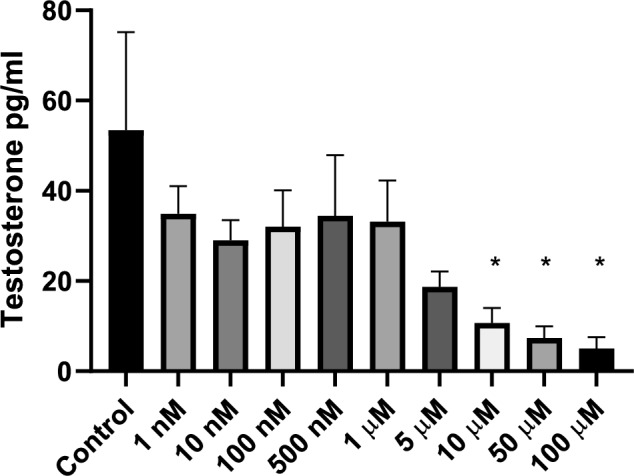
DBP exposure significantly reduced the testosterone production in the 3D testicular co-culture. The testosterone level in media was measured by ELISA. Values represent mean ± SEM from three experiments. Each treatment condition was performed in three wells per experiment. Statistically significant differences from control are indicated as *p < 0.05 (one-way ANOVA followed by Dunnett’s multiple comparison test)

The testosterone level was analyzed by ELISA in the culture media after 7 days of DBP exposure. DBP treatment induced a significant concentration-dependent decrease in the levels of testosterone of 80% (*p* = 0.029), 86% (*p* = 0.017) and 91% (*p* = 0.011) at 10 µM, 50 µM and 100 µM, respectively (Fig. 3). Since DBP treatment at 50 µM and 100 µM also caused a significant decrease in cell viability, these two concentrations were excluded from further experiments.

### DBP exposure significantly reduced the level of key testicular proteins in the testosterone biosynthesis

**Fig. 4:**
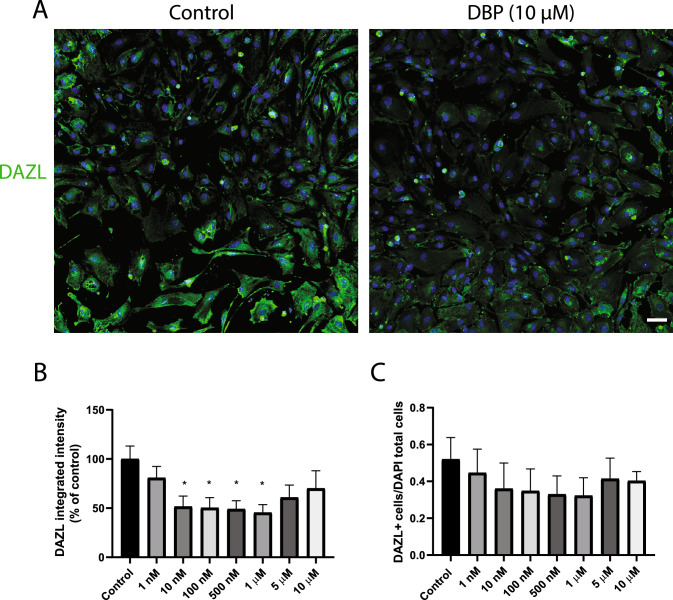
DBP effects on the germ-cell marker DAZL in the 3D testicular co-culture. Immunostaining and high-content imaging analysis of DAZL following 7-day exposure to various DBP concentrations or solvent control (0.1% DMSO). **A** Representative composite images of testicular cells stained with DAPI (blue) and DAZL (green) after exposure to solvent control or 10 µM DBP. **B** DAZL levels measured as integrated intensity. **C** Number of DAZL-positive cells per image divided by the total cell count indicated by DAPI-stained nuclei. Values represent mean ± SEM from three experiments. Each treatment condition was performed in three wells per experiment. Statistically significant differences from control are indicated as follows: *p < 0.05 (one-way ANOVA followed by Dunnett’s multiple comparison test). Scale bar = 100 µm

**Fig. 5:**
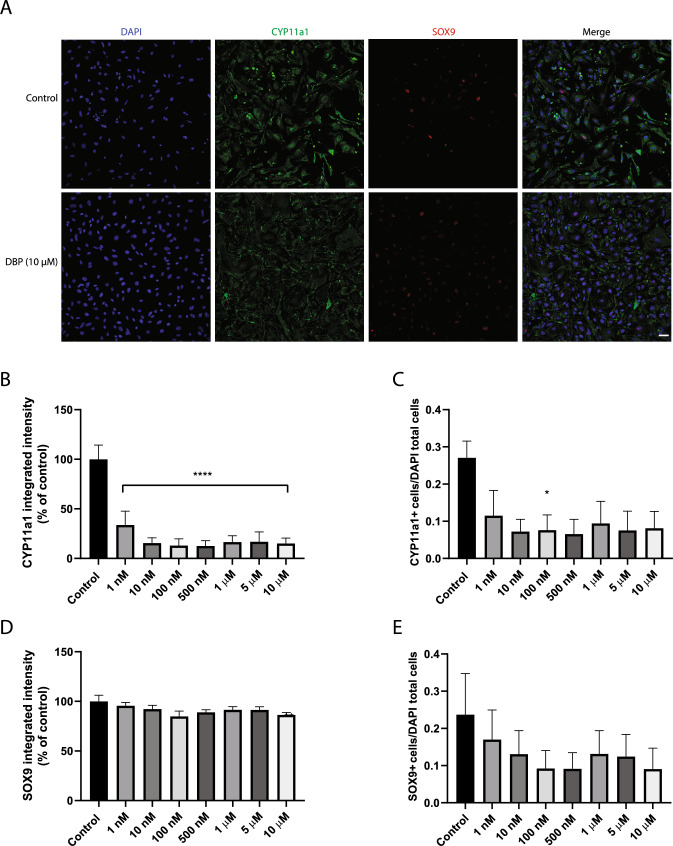
DBP effects on Leydig and Sertoli cell markers in the 3D testicular co-culture. Double-immunostaining and high-content imaging analysis of CYP11A1 and SOX9 following 7-day exposure to various concentrations of DBP or solvent control (0.1% DMSO). **A** Representative composite images of testicular cells co-stained with DAPI (blue), CYP11A1 (green) and SOX9 (red) after exposure to solvent control or 10 µM DBP. **B** CYP11A1 levels measured as integrated intensity. **C** Number of CYP11A1-positive cells per image divided by the total cell count indicated by DAPI-stained nuclei. **D** SOX9 expression level measured as integrated intensity. **E** Number of SOX9-positive cells per image divided by the total cell count indicated by DAPI-stained nuclei. Values represent mean ± SEM from three experiments. Each treatment condition was performed in three wells per experiment. Statistically significant differences from control are indicated as *p < 0.05, **p < 0.01, ***p < 0.001 and ****p < 0.0001 (one-way ANOVA followed by Dunnett’s multiple comparison test). Scale bar = 100 µm

**Fig. 6:**
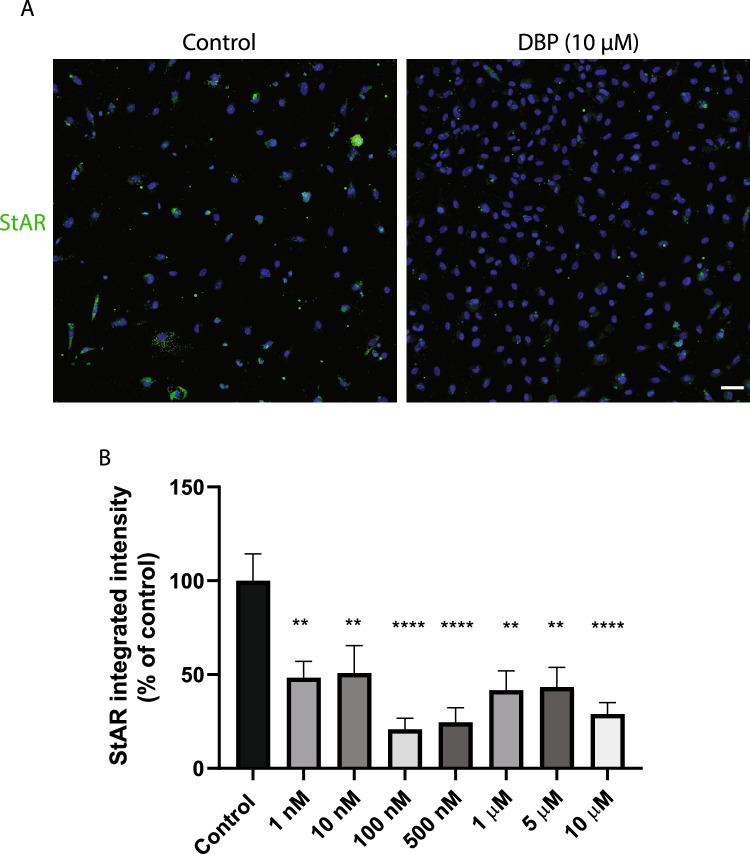
DBP effects on steroidogenic acute regulatory (StAR) protein levels in the 3D testicular co-culture. Immunostaining and high-content imaging analysis of StAR following 7-day exposure to various concentrations of DBP or solvent control (0.1% DMSO). **A** Representative composite images of testicular cells stained with DAPI (blue) and StAR (green) after exposure to solvent control or 10 µM DBP. **B** StAR levels measured as integrated intensity. Values represent mean ± SEM from three experiments. Each treatment condition was performed in three wells per experiment. Statistically significant differences from control are indicated as **p < 0.01, ***p < 0.001 and ****p < 0.0001 (one-way ANOVA followed by Dunnett’s multiple comparison test). Scale bar = 100 µm

Using immunofluorescence staining of cell-specific markers and automated high-content imaging, we confirmed the presence of the three major testicular cell types: germ cells (DAZL-positive cells), Sertoli cells (SOX9-positive cells), and Leydig cells (CYP11A1-positive cells) in the 3D testicular co-culture. DBP caused a 48–55% decrease in DAZL protein levels, after exposure to 10 nM, 100 nM, 500 nM and 1 µM, compared to control (p < 0.05) (Fig. 4A & B). There was a similar trend toward a decrease in the ratio of DAZL-positive cells divided by the total cell count (Fig. 4C). All DBP concentrations tested induced a significant 66–85% reduction in CYP11A1 levels compared to the control (*p* < 0.0001, Fig. 5A & B). The relative count of CYP11A1-positive cells also decreased, demonstrating a potential DBP-induced decline in the Leydig cells number in the 3D co-culture (Fig. 5C). DBP induced no effect on the protein levels of SOX9 (Fig. 5A, D & E). The immunostaining of the steroidogenic regulator StAR was significantly decreased (49–79%) by all tested DBP concentrations compared to the control *(p* < 0.05; Fig. 6A & B).

### The 3D testicular co-culture captured the development of Sertoli tight and adherens junctions

**Fig. 7:**
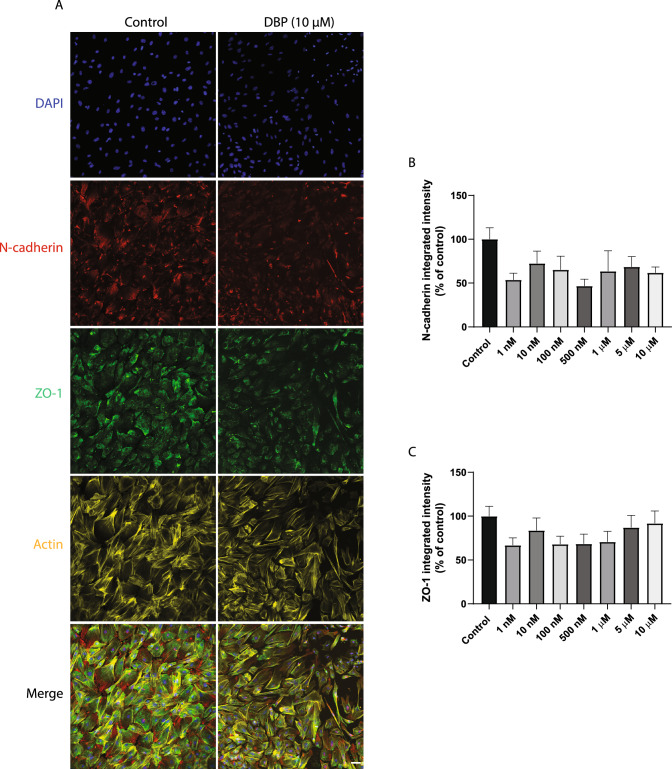
Formation and immunolocalization of tight and adherens junction proteins in the testicular 3D co-culture. Triple-immunostaining and high-content imaging analysis of N-cadherin, ZO-1 and F-actin after 7-day exposure to various concentrations of DBP or solvent control (0.1% DMSO). **A** Representative images of testicular cells triple-stained with DAPI (blue), N-cadherin (red), ZO-1 (green) and F-actin (yellow) after exposure to solvent control or 10 µM DBP. **B** N-cadherin levels measured as integrated intensity. **C** ZO-1 levels measured as integrated intensity. Values represent mean ± SEM from three experiments. Each treatment condition was performed in three wells per experiment. The statistical analysis revealed no significant differences between the DBP-exposed cells and control group (one-way ANOVA followed by Dunnett’s multiple comparison test). Scale bar = 100 µm

Another advantage of this 3D testicular co-culture model is the possibility to study the development of the essential junction proteins, which are critical for the blood–testis barrier. Using immunofluorescence staining and automated high-content imaging, the critical adherens and tight junction proteins N-cadherin and ZO-1 were captured. Both N-cadherin and ZO-1 appeared to be downregulated in DBP-exposed cells compared to control. However, the decrease did not reach statistical significance (Fig. 7A–C).

### DBP exposure altered the levels of proteins involved in testosterone synthesis

**Fig. 8:**
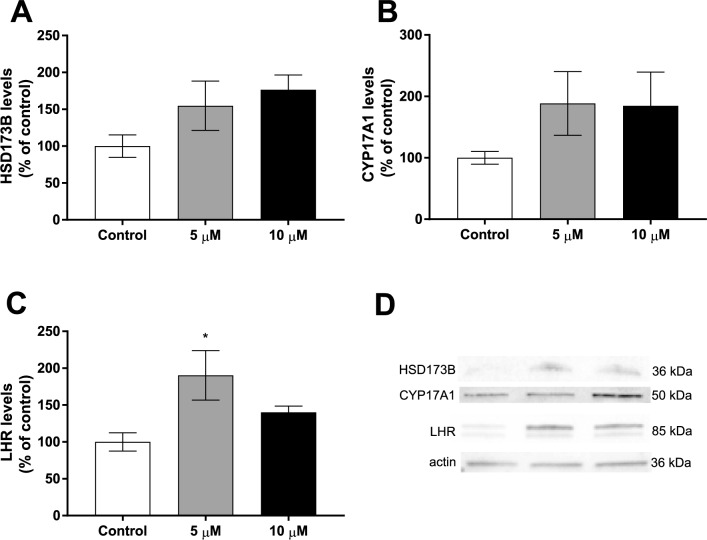
DBP effects on steroidogenic protein levels were measured by western blot in the 3D testicular co-culture exposed to 0.1% DMSO (solvent control), 5 µM or 10 µM DBP for 7 days. **A** HSD173B levels. **B** CYP17A1 levels. **C** LHR levels. Values represent mean ± SEM from six replicates per treatment. **D** Representative blots for each protein are shown. β-actin was used as a loading control. The statistically significant differences from control are indicated as follows: *p < 0.05 and **p < 0.01 (one-way ANOVA followed by Dunnet’s multiple comparison test)

To further characterize the mechanisms underlying the decreased testosterone production, the levels of HSD173B, CYP17A1 and Luteinizing hormone receptor (LHR) were measured by western blot after exposure to 5 µM and 10 µM DBP (Fig. 8A–D). Neither HSD173B nor CYP17A1 levels were altered (Fig. 8A–B). However, the levels of LHR was increased in the 5 µM DBP-exposed cells compared to control (90%, *p* < 0.05) (Fig. 8C).

## Discussion

The development of new approach methodologies (NAMs) that include application of new technology can accelerate the discovery of specific modes of action and allow better prediction of toxicological potential in chemical-risk assessment and drug development (Karlsson 2023; Karlsson et al. 2021; Westmoreland et al. 2022). There is a significant need for alternative testing methods for reproductive toxicants that are practical, fast, cost-effective and generate relevant high-quality data compared with traditional in vivo methods. Due to the sensitivity of the early childhood genital development, which can lead to lifelong implications, there is an unmet demand for relevant in vitro toxicological models to better understand the testicular toxicity pathways (Quinton et al. 2017; Renault et al. 2020). Here, we developed an in vitro 3D testicular cell culture model, obtained by cultivating neonate mouse spermatogonia together with somatic cells, including proliferating Sertoli and Leydig cells that captured reprotoxic effects of DBP at concentrations as low as 1 nM.

A similar in vitro model derived from 5-day-old Sprague–Dawley rat pups has previously been established (Wegner et al. 2013). However, an advantage of a cell model based on mouse testis is the ability to study genetic factors by collecting tissues from transgenic mice. In addition, to efficiently screen for chemical reproductive toxicants and underlying toxicity pathways, high throughput screening (HTS) methods are favored. We have therefore enhanced the in vitro model throughput  by optimizing the cell seeding into 96-well plates compared to the previous protocol where cells were seeded in culture dishes limiting the number of samples (Wegner et al. 2013). This allowed the use of automated high-content imaging and quantification of fluorescence intensities to improve the time efficiency and include assessment of further parameters, such as the development of Sertoli cell-tight junctions.

DBP was used as a model compound to illustrate the utility of the 3D testicular in vitro model for reproductive toxicity testing. DBP has been reported to be a male reproductive toxicant with anti-androgenic endocrine disrupting activity in both fetal and adult male gonads. In the context of prenatal and adult exposure, its mechanism of action is characterized by multiple cellular processes, including impaired Leydig cells steroidogenesis and Sertoli cell function, disrupted seminiferous cord formation and maturation, impaired germ-cell survival, and formation of multinucleated germ cells (Källsten et al. 2022a, 2022b; Lee et al. 2004; Mahaboob Basha and Radha 2017; Saillenfait et al. 1998; Zhang et al. 2004). Here, the impact of 7-day exposure to a wide range of DBP concentrations (1 nM–100 µM) was investigated using the 3D testicular co-culture, capturing in vivo biologic plausible cell signaling pathways and hormone production. The results revealed that exposure to 10 µM, 50 µM, and 100 µM DBP significantly reduced the testosterone production. The levels of steroidogenic regulator StAR and the steroidogenic enzyme CYP11A1 were decreased already at the lowest DBP concentration tested (1 nM), demonstrating upstream effects in the testosterone biosynthesis pathway. DBP exposure also decreased DAZL levels at concentrations as low as 10 nM. DAZL is a germ cell-specific RNA binding protein that plays a crucial role in regulating the post-transcription expression of germline genes (Zagore et al. 2018). Previous studies have shown that disruption in *Dazl* expression can negatively impact the initiation of meiosis and differentiation of germ cells. *Dazl* knockout mice resulted in germ-cell apoptosis (Ruggiu et al. 1997; Zagore et al. 2018). Therefore, this suggests that the DBP-induced downregulation of DAZL may disrupt germ-cell development and differentiation. We have previously shown in vivo that 5 weeks adult exposure to 10 mg/kg/day DBP results in an increase in DAZL protein levels in mice, when measured one week after the last DBP dose was administered (Källsten et al. 2022a). In contrast, 2 weeks adult exposure to the DBP metabolite, MBP, at a dose of 200 mg/kg has been reported to downregulate *Dazl* mRNA expression in mice (Du et al. 2017). The discrepancies in the literature and between our in vitro and in vivo data may be attributed to differences in testicular germ-cell regulation at different developmental stages, as the current model uses newborn mice compared to fully mature adult mice. Variations in the concentrations and exposure times used, time for collecting and measuring effects in tissues, as well as the contribution of DBP metabolic products in the in vivo systems could also play a role in the differential DBP exposure outcomes observed.

Mechanistically, the demonstrated decrease in testosterone production appears to be secondary to the observed decrease of the steroidogenic regulatory proteins StAR and CYP11A1 (Borch et al. 2006; Gray et al. 2000). However, the decrease in testosterone levels could also be partly due to the DBP-induced decrease in Leydig cell number (Ikeda et al. 1994). As DBP did not decrease the cell viability at concentrations up to 10 µM in our 3D testicular co-culture model, it is likely that the decrease in the Leydig cell number is due to the decrease in proliferation and differentiation. For testosterone formation, StAR expression is essential for the translocation of cholesterol to the inner mitochondrial membrane, which is a rate-limiting and -determining step in Leydig cell steroidogenesis (Payne and Hales 2004). Cholesterol is then metabolized into pregnenolone by the cholesterol side-chain cleavage enzyme CYP11A1. Pregnenolone subsequently enters the smooth endoplasmic reticulum, where testosterone is synthesized, through a series of enzymatic reactions (Payne and Hales 2004). Interestingly, StAR knockout mice suffer from adrenocortical and gonadal steroid deficiency, with a similar phenotype being described in CYP11a1 null mice (Caron et al. 1997; Hu et al. 2002). As both StAR and CYP11A1 affect the pathway of cholesterol utilization, their deficiencies result in accumulation of cholesterol, leading eventually to Leydig cell damage and tissue atrophy (Hu et al. 2002). Exposure to different classes of phthalates, including DBP has been shown to downregulate StAR and CYP11A1 among other steroidogenesis-related proteins in fetal Leydig cells (Lehmann et al. 2004; Wang et al. 2019). For instance, 5 days in utero exposure to DBP (500 mg/kg/day) resulted in significant reduction in testosterone production and a decrease in StAR mRNA expression in fetal testis. Interestingly, the DBP effects were reversible upon discontinuation of DBP exposure for 2 days (Thompson et al. 2004). Despite the well-documented effects of DBP on the upstream steroidogenic proteins, the direct mechanism behind the DBP-induced downregulation of StAR and CYP11A1 is yet to be explored. The DBP-induced decrease in testosterone in our in vitro model is consistent with the numerous in vivo studies that have reported more than 40% decrease in fetal testosterone levels following in utero exposure to high DBP concentrations (≥ 100 mg/kg body weight/day) (Johnson et al. 2007; Johnson et al. 2011; van den Driesche et al. 2012; Zhang et al. 2004). The reduced fetal testosterone production is suggested to be a consequence of altered Leydig cell differentiation and function (Borch et al. 2006; Fisher et al. 2003; Gray et al. 2000).

Testosterone production is differentially regulated in neonates and adults due to developmental variations in hormonal regulation (Geissler et al. 1994; Weinbauer et al. 2010). Although testosterone is often assessed in the context of adult male fertility, neonatal testosterone production, known as the minipuberty or postnatal testosterone surge, appears to have implications on future reproductive function. In neonatal mice, although this postnatal hormone surge is transient and followed by a quiescent period until the onset of puberty, it has been suggested as a critical programming window with long-term consequences (Corbier et al. 1992; Renault et al. 2020). In humans, the minipuberty is documented in infants and has been reported to play significant roles in the sexual differentiation and maturation of male genitalia important for future fertility (Quinton et al. 2017; Renault et al. 2020). Minipuberty is believed to be driven by human chorionic gonadotropin (hCG), which acts similarly to the luteinizing hormone (LH) and can activate Leydig cells, promoting testosterone synthesis in neonates (Choi and Smitz 2014). In addition, a transient activation of hypothalamic-pituitary–gonadal (HPG) axis has been reported in neonates, although the HPG axis is not fully developed (Bizzarri and Cappa 2020).

Similar to our previous in vivo DBP exposure studies (Källsten et al. 2022a), the in vitro DBP exposure model demonstrated an increase in LHR expression in Leydig cells. Notably, the decreased testosterone production in the in vitro system could also be due to impaired Sertoli-Leydig cells interaction, as testosterone levels have been shown to both decline when Leydig cells are cultured alone, and increase when Leydig cells co-culture with Sertoli cells (Lejeune et al. 1998). This indirect influence of Sertoli cell on testosterone synthesis may be via steroidogenic factors secretion, such as locally produced growth factors, cytokines and paracrine mediators, which can enhance Leydig cell steroidogenic activity and stimulate testosterone secretion in vitro (Boujrad et al. 1995; Lejeune et al. 1998).

Overall, the impact of DBP, and other chemicals, on the development of tight junction proteins is understudied compared to effects on testosterone production. It is therefore important to develop suitable methods that allow this kind of studies. Here, we were able to show the robustness of our in vitro 3D testicular cell-culture model for studying two junction proteins that are main components of Sertoli-Sertoli junctions, N-cadherin and ZO-1 (Sharpe et al. 2003; Vogl et al. 2008). These junctional proteins, among others, constitute the basis for the blood–testis barrier structure. Thereby, dividing the seminiferous epithelium into two compartments, a basal compartment that comprises immature germ cells and an adluminal (apical) compartment that contains more mature germ cells (Mruk and Cheng 2015). It comprises of specialized cell junction complexes of occluding tight junctions, like ZO-1 and adherens junctions, such as N-cadherin coexisting with gap junctions and ectoplasmic specializations. The function of blood–testis barrier is to protect the developing germ cells from the circulatory harmful substances and lymphatic systems and, together with local immune suppression, to provide an immune-privileged microenvironment for the completion of meiosis (Mruk and Cheng 2015; Vitale et al. 1973). Several studies have shown that the structure and integrity of tight junction proteins are adversely affected by in vivo exposure to different classes of phthalates (de Freitas et al. 2016; Kleymenova et al. 2005; Sekaran et al. 2015; van den Driesche et al. 2015). In our study, in vitro exposure to DBP had no significant effect on the junction proteins tested, N-cadherin and ZO-1. This lack of effect could be attributed to differences in experimental models, timing and duration of exposure, and possibly the mechanism of action of the phthalates used compared to DBP.

In summary, this in vitro 3D testicular co-culture model provides an opportunity to study chemical effects on hormone production and unique postnatal cellular pathways. Together with high-content imaging using multiplexed fluorescence markers, this cell model provides a feasible screening tool to identify potential toxicants that may induce cellular responses involved in germ-cell development, Leydig cell steroidogenesis function or inter-Sertoli cell junctions. This approach can generate both quantitative and qualitative measurements to characterize the impact of chemicals on testicular cell physiologic function and subcellular structures, in addition to profiling the cell phenotypes in the 3D testicular co-culture. In line with the literature, we showed that the developing Leydig cell function and number are significantly vulnerable to low concentrations of DBP. This indicates that the testicular co-culture is a biologically relevant model to explore molecular mechanisms involved in impaired testosterone synthesis. Furthermore, because there is much emphasis on early childhood exposures and effects on the genesis of adult disease, such a developmental 3D testicular co-culture model could also be useful to characterize for example epigenome modulation during the sensitive postnatal development.

## Data Availability

Data will be made available on request.
